# Sexual Function, Physical Activity, Mean Amplitudes and Maximal Voluntary Contraction of Pelvic Floor Muscles Are Related to Handgrip Strength: A Cross-Sectional Study

**DOI:** 10.3390/healthcare11010129

**Published:** 2022-12-31

**Authors:** Natália de Souza Duarte, Kayonne Campos Bittencourt, Cleuma Oliveira Soares, Clicia Raiane Galvão Ferreira, Wenderk Martins Soares, Victória Brioso Tavares, Amanda Suzane Alves da Silva, Caren Heloise da Costa Priante, Pablo Fabiano Moura das Neves, Givago da Silva Souza, Fabiana de Campos Gomes, Erica Feio Carneiro Nunes, Cibele Nazaré Câmara Rodrigues, João Simão de Melo Neto

**Affiliations:** 1 Institute of Health Sciences, Federal University of Pará (UFPA), Belém 66075-110, PA, Brazil; 2 CAFISIO Mulher, Belém 66055-190, PA, Brazil; 3 State University of Pará (UEPA), Belém 66075-110, PA, Brazil; 4 Ceres Faculty of Medicine (FACERES), São José do Rio Preto 15090-305, SP, Brazil; 5 Clinical and Experimental Research Unit of the Urogenital System (UPCEURG), Institute of Health Sciences of Federal University of Pará, Mundurucus street, 4487, Guamá, Belém 66073-000, PA, Brazil

**Keywords:** pelvic floor, muscle strength dynamometer, aging, electromyography

## Abstract

Pelvic floor musculature assessment methods are generally invasive, subjective, and technologically expensive. Therefore, there is a need to identify other methods that can predict changes in the function of these muscles. This study aimed to verify whether the levels of strength and myoelectric activity of pelvic floor muscles (PFM) can be related to handgrip strength (HGS), to ensure faster and earlier identification of possible dysfunctions of this musculature. Furthermore, we verified whether these variables vary across different age groups. This was a cross-sectional observational study involving 44 healthy women. The women were divided into two groups: the young (18–35 years) and middle-aged (36–55 years) adult groups. Social, anthropometric, and clinical data were collected from the participants, and a functional assessment of their PFM was performed by bidigital palpation, electromyographic biofeedback (sEMG), and HGS (using a dynamometer). The levels of physical and sexual activity were measured using the International Physical Activity Questionnaire (IPAQ) and Sexual Quotient–Female version (SQ-F) questionnaire. There were no differences in HGS, power/pressure, sEMG, SQ-F score, or IPAQ score between the two groups (*p* > 0.05). Moderate correlation (r = 0.601; *p* = 0.019) was observed during multivariate analysis. HGS is related to mean amplitudes (*p* = 0.123), MVC (*p* = 0.043), sexual function (*p* = 0.049), and physical activity (*p* = 0.004). We therefore conclude that there were no differences between HGS and PFM strength in young adult and middle-aged women. Furthermore, HGS is related to the PFM functionality, sexual function, and physical activity.

## 1. Introduction

The pelvic floor is formed by muscles, ligaments, and fascia that have sphincteral, sexual, postural, and supportive functions. Its muscular component is made up of deep and superficial layers responsible for sphincter control [1,2]. However, like all muscles, they are subject to changes in functionality caused by various factors such as aging, pregnancy, traumatic births, and poor urinary habits [3].

In the event of an alteration in its functionality, the evaluation of the pelvic floor muscles (PFM) will be of paramount importance for the choice and adoption of an effective treatment plan. The International Continence Society recommends that assessments be performed by visualization, bidigital palpation, and electromyography studies [4,5]. With the exception of visualization, these methods are invasive [5] and cannot be performed in all circumstances due to lack of adequate infrastructure or equipment. Therefore, there is a need for reliable, low-cost, and easy-to-apply tools that can predict the strength of these muscles.

The measurement of handgrip strength (HGS) by dynamometry has been reported to be a good indicator of overall muscle strength [6,7] and can be used as an auxiliary diagnostic tool in PFM disorders. Previous studies have related handgrip weakness to geriatric incontinence [8,9] and have shown better PFM functionality results in women who practice physical activity when compared to sedentary women (suggesting a relationship between overall muscle strength and PFM strength) [10,11]. However, even though there has been this great need for tools that facilitate the functional assessment of PFMs, no studies have assessed HGS as a possible predictor of pelvic muscle function. Validation of this type of instrumentation is essential for clinical practice, especially considering the presence of pelvic floor muscle dysfunction even in physically active women [A,B].

Furthermore, it is important to take into consideration the fact that PFMs can influence female sexual function. However, few studies have assessed sexuality in women with pelvic floor disorders, despite the close association between pelvic floor disorders and sexual dysfunctions. Some recent studies have shown that women with sexual dysfunctions have lower PFM strength than those without dysfunctions [12,13].

Considering these factors, it is important that new PFM assessment tools be studied to ensure a faster and earlier identification of possible dysfunctions of this musculature. Therefore, the aim of this study was to verify whether handgrip strength and PFM strength and bioelectrical activity change in young adult and middle-aged women. In addition, we wanted to verify whether the strength and bioelectrical activity of PFMs are related to handgrip strength. Finally, we sought to verify whether the levels of physical activity and sexual function interfered with this relationship. In this sense, our initial hypotheses were: there are differences in HGS between young and middle-aged female adults, HGS is related to PFM strength in healthy women, and physical activity and sexual function modulate this relationship.

## 2. Methods

### 2.1. Study Design

This was a cross-sectional observational study with descriptive and inferential analyses.

### 2.2. Setting and Period of Study

The study was conducted using data obtained from a private clinic specialized in the field of women’s health in the city of Belém, PA, Brazil, during the year 2021.

### 2.3. Population

The study was conducted on young (age range: 18–35 years) and middle-aged adult women (age range: 36–55 years) [14].

### 2.4. Eligibility Criteria

Women aged between 18 and 55 years who signed the Free and Informed Consent Form were included. Those who had conditions (such as a fracture, pain, tingling, or numbness) in their hand which prevented them from performing any of their daily living activities in the last 12 months; pregnant women; those in the immediate postoperative period; those diagnosed with a neoplasia or undergoing cancer treatment; those with neurological disorders; those with a prolapse of grade >2 during the Valsalva maneuver (in accordance with the modified Baden and Walker classification) [15]; those with active urinary or vaginal tract infections; and those with any other condition that could alter the results of the present study, as well as those with incomplete data in the evaluation form, were excluded.

### 2.5. Sampling

A non-probability convenience sampling method was used to select patients.

### 2.6. Sample Size

The initial sample comprised 44 healthy women. Concerning sample size calculation, applied analysis was used to verify whether HGS was related to PFM function. The chosen sample size considered a minimum of 20 participants with an actual power of 0.806, based on the R^2^ of two predictors (“hip extensor strength” and “pelvic floor muscle endurance”) determined from the study by Hwang et al. [16]. The effect size was measured to be 0.449, with a *p*-value < 0.05 (for the two-tailed analysis) and an α error of 0.05 and β error of 0.2. The sample size was calculated using the software G* Power (version 3.1.3; University of Trier, Trier, Germany).

### 2.7. Data Collection and Variables

All patients were evaluated by a single physical therapist. The social, anthropometric, and clinical data of all the women were collected using a form developed by the authors.

The social variables collected were age (years), race (Black, Brown, White, Yellow), education (elementary school, secondary school, or higher education), marital status (single, stable union, or divorced), and family income (<1 minimum wage; >1 and ≤2 minimum wages; >3 and ≤4 minimum wages; and >4 minimum wages). The minimum wage considered was the Brazilian one (1.212 reais).

The anthropometric variables collected were weight (kg), height (m), and body mass index (BMI) (kg/m^2^).

The clinical variables collected were number of pregnancies, number of abortions, ongoing hormone replacement therapy, active sexual life in the last three months, and the presence or not of urinary and anal dysfunctions. The urinary disorders analyzed were nocturia, preventive urination, urinary tenesmus, urinary tract infections (in the last three months), enuresis, urinary urgency, urge urinary incontinence, stress urinary incontinence, and dysuria. The anal dysfunctions studied were constipation (ROMA III) [17]; fecal urgency, fecal tenesmus, soiling, fecal leakage, and hemorrhoids. The Rome III criteria for the diagnosis of constipation (anal dysfunction) were applied.

For the functional assessment of the pelvic floor, electromyographic biofeedback and bidigital palpation were used to evaluate the level of strength (power/pressure) of the pelvic floor muscles. Hand grip strength was evaluated by dynamometry.

### 2.8. Primary Outcomes

The primary outcomes considered in the present study were the level of HGS and the levels of strength and bioelectrical activity of the PFMs in healthy women.

#### 2.8.1. Handgrip Strength (HGS)

HGS was evaluated using a previously calibrated hand hydraulic dynamometer device (SH5001). The volunteers were positioned in a seated position on a chair with a backrest, with the forearm resting on the arm of the chair, the shoulder abducted and neutrally rotated, the elbow flexed at 90°, and the forearm and wrist in neutral position. The technique consisted of performing a maximum grip with the dominant hand. The technique was repeated three times, with a one-minute rest between the attempts, and the highest score was considered [6,8].

#### 2.8.2. Level of Strength of the Pelvic Floor Muscles

For this assessment, the participant remained in the supine position, with the hip and knee flexed, and the two feet supported on the stretcher and positioned apart from each other. The muscle contraction capacity of the pelvic floor muscles was assessed using the PERFECT (P = power or pressure, E = endurance, R = repetitions, F = fast contractions, and ECT = every contraction timed) scheme [18], an evaluation method created to qualify the main components of pelvic muscle contractility. We only used the P component (power/pressure), whose scoring varies from 0 to 5 (0 = no contraction; 1 = flicker; 2 = weak; 3 = moderate; 4 = good (with lift); 5 = strong). The P component is used to evaluate perineal muscle strength through digital palpation during voluntary contraction. We used the modified Oxford grading system [19,20].

#### 2.8.3. Bioelectrical Activity of the Pelvic Floor Muscles

Surface electromyography (sEMG) was performed to evaluate the bioelectrical activity of the muscles. We used the new Miotool biofeedback electromyography machine (Miotec Equipamentos Biomédicos Ltd. a Porto Alegre, RS, Brazil) which has eight channels and a resolution of 16 bits. For data processing, a software in the biotrainer modality inserted into the MiotecSuite 1.0.1108 (Miotec^®^) was used. This equipment captures the electrical activity of the muscle in microvolts (µV) through an electromyography sensor placed on the patient’s lateral malleolus. The volunteers were instructed to remain in the supine position with hips flexed and abducted and knees flexed; an intracavitary vaginal electrode and a reference electrode positioned on the stretcher were then placed [21]. Next, the therapist gave a verbal command to the patient to contract her pelvic floor muscles while he measured the maximum voluntary contraction (MVC) of the pelvic floor muscles. The peak contraction and average contraction amplitude were measured in the range of 0 to 10 s in raw and normalized data (%MCV).

### 2.9. Secondary Outcomes

The secondary outcome considered was the association between the primary outcomes and the level of physical activity and sexual function in the women.

#### 2.9.1. Physical Activity

The short version of the International Physical Activity Questionnaire (IPAQ) [22] was used to measure the level of physical activity. This questionnaire estimates the weekly amount of time spent in physical activities of light, moderate, and vigorous intensities. The questions in the questionnaire make reference to activities carried out during the week prior to the application of the questionnaire, dividing and conceptualizing the level of activity into the following categories: Sedentary, Insufficiently Active (Insufficiently Active A and Insufficiently Active B), and Active.

In addition, data on self-reported physical activity were collected.

#### 2.9.2. The Sexual Quotient—Female Version (SQ-F) Questionnaire

This questionnaire is composed of 10 questions concerning several domains of women’s sexual activity such as desire, arousal, and orgasm, as well as their respective psychophysical correlates. The questions were based on the patients’ sexual experiences of the last six months, and their responses were rated on a scale ranging from 0 to 5. The scores were added and multiplied by two, and then recorded on a scorecard. High values indicate better sexual performance/satisfaction (82–100 points = good to excellent, 62–80 points = fair to good, 42–60 points = unfavorable to fair, 22–40 points = poor to unfavorable, 0–20 points = null to bad) [23].

### 2.10. Statistical Analysis

Descriptive statistical analysis was conducted to compute the frequency (absolute and relative (%)), the means with standard deviation (parametric), or the medians with interquartile ranges (IQR) (non-parametric) for each age group. The unpaired t-test (parametric), Mann–Whitney U test (non-parametric), and chi-square test (χ^2^) (categorical data) were used to verify if there were differences (with respect to the social, anthropometric, and clinical variables) between the two age groups. Multivariate linear regression analysis was used to verify the predictive value of HGS using the power/pressure results and the electromyography parameters (MVC (µv); peak (0–10 s); normalized peak (% MCV); mean amplitudes (0–10 s); normalized mean amplitudes (%MVC)), SQ-F and IPAQ; and the variables used in the HGS model. All statistical analyses were performed using the software Statistical Package for Social Sciences (SPSS for Windows, v21.0; IBM) with the α value set at 0.05.

## 3. Results

**Figure 1:**
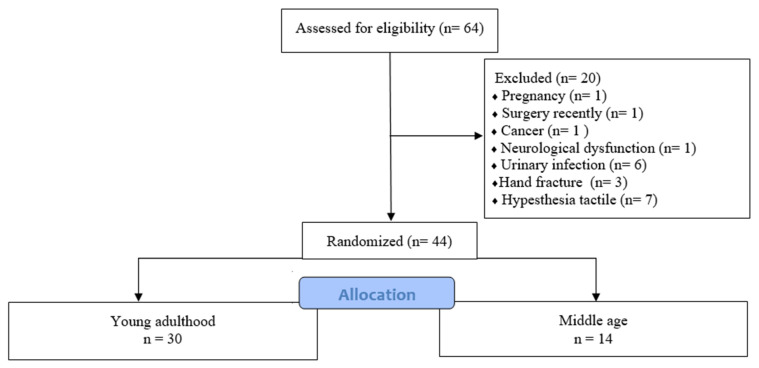
Flowchart of sample eligibility.

The 44 healthy women were divided into the following groups: young adulthood (18–34 years) (*n* = 30) and middle age (35–55 years) (*n* = 14). A flowchart of the sample eligibility is shown in Figure 1.

The mean age differed significantly between the two age groups (young and middle age) (*p* < 0.0001) (Table 1). In terms of marital status, young adult women were predominantly single, while middle-aged women were predominantly divorced (*p* < 0.0001) (Table 1). Young adult women had higher height (*p* = 0.045) (Table 1). The other social and anthropometric factors were not different.

The numbers of pregnancies (*p* < 0.0001) and abortions (*p* = 0.044) were higher in middle-aged women (Table 2). The other clinical variables were not different.

The variables HGS, power/pressure, sEMG records, SQ-F, and IPAQ were analyzed by age group (young adulthood and middle age), and no statistically significant difference was found in the comparisons between groups (*p* > 0.05) (Table 3), demonstrating the equivalence of the data from both age groups. Therefore, these data were integrated to evaluate whether HGS can be used as a functional predictor of the strength of PFMs.

Before building the multiple linear regression model, the hypotheses were tested. The result of the Durbin–Watson test was 1.976, and it was within the acceptable range of [1.5; 2.5] to demonstrate the independence of residuals. The Cook’s distance (mean: 0.032 ± 0.063) for each observation was less than 1, so there were no outliers in the dataset that negatively affected the estimation of the coefficients. In sequence, the values of VIF and Tolerance obtained for each independent variable were tested, and the Peak (0 to 10 s) variable was excluded from the final model. The values of VIF (<10) and Tolerance (>0.2) of the final model are shown in Table 4, so the absence of multicollinearity was verified.

**Figure 2:**
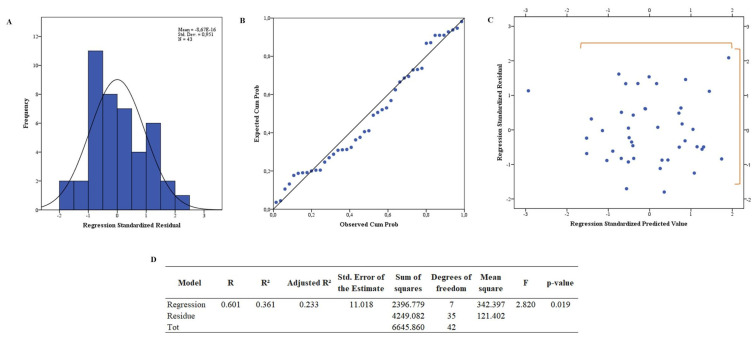
Gaussian distribution (**A**), normal P–P plot of standardized residual (**B**), plot of “standardized residuals” against the “standardized predicted value” (**C**), model summary and Fisher’s exact test (**D**) of the final model.

Figure 2 shows the Gaussian distribution (Figure 2A) and the P–P plot (Figure 2B), in which a comparison of the “observed probability” versus “expected probability” is used to test the normal distribution of the residuals. As can be seen in Figure 2A, the graph shows a parametric distribution, and in Figure 2B, the points are quite close to the line. There are a few outliers, but they have been shown not to affect the quality of the coefficient estimate. In fact, the Cook’s distance was calculated for each point, and the maximum value was 0.31, which is well below the required threshold value of 1.

Figure 2C shows the graph of “standardized residuals” against the “standardized predicted value” used to verify that the variance of the residuals is constant. The variance of residuals was constant across predicted values.

In fact, Figure 2D shows that the analysis of variance is significant, i.e., there is indeed a linear dependence between the dependent variable and the regressor variable (*p*-value  <  0.05). Then, the MLR model was implemented.

The coefficient of correlation (R) was greater than 0.6, so it can be considered a moderate preliminary model to represent the problem. Table 4 shows the coefficients of the model and the results of the t-test, used to study the significance of the regression coefficients (β).

The *p*-value was less than 0.05 for the mean amplitudes (0 to 10 s), MVC (µv), SQ-F, and self-declared physical activity. Among these variables, the self-declared physical activity and MVC (µv) have the highest coefficient.

## 4. Discussion

PFM assessment tools are generally invasive, subjective, and technically expensive. Therefore, there is a need to find other methods that can contribute to identifying possible changes in the function of these muscles. Therefore, the aim of this study was to verify whether the strength and bioelectrical activity of the pelvic floor muscles can be related to HGS and if there are differences between these changes in different age groups.

Initially, we analyzed social, anthropometric, and clinical variables in young and middle-aged women. In this analysis, we found differences in marital status, which was predominantly “divorced”, and a higher number of pregnancies and miscarriages in middle-aged women. These results are to be expected with advancing age.

Our results showed no differences between young and middle-aged women in HGS, strength, and bioelectrical activity of PFMs. Regarding HGS, the literature indicates that there is an age-related decline in women from the age of 50 years [24], which is consistent with our result, where we analyzed middle-aged women with an average age of 43 years. Regarding the functionality of PFMs, it has been demonstrated by vaginal palpation, manometric values, and bioelectrical activity that there are no changes in the pattern in different age groups between 18 and 69 years [25], which was also observed in our study. Therefore, our results show that the age groups of women do not differ with respect to our primary outcomes, confirming previous findings in the literature [26,27]. Thus, all women were included in subsequent analyses.

No statistically significant relation was found between HGS and PFM strength (measured by vaginal palpation); this is confirmed by a study by Swenson [26], who found no correlation between HGS strength and PFM strength (measured by Kegel augmentation strength) when comparing groups of young (<40 years old) and older (≥70 years old) nulliparous women. On the other hand, Bag Soytas et al. [27] demonstrated a correlation between HGS, PFM strength, and perineometer measurements in women with urinary incontinence. This divergence from our results could be due to differences in the methods used to assess PFM strength and the numbers of participants in the studies. Although our assessment was performed using protocols from the literature and by an experienced evaluator, it was emphasized that vaginal palpation (even when performed by an experienced evaluator) is not the best tool for a researcher because of the subjectivity of its interpretation.

In the present study, it was found that HGS can be related to PFM bioelectrical activity (mean amplitudes and MVC). PFM function can be measured by bioelectrical activity—another study has already demonstrated this relationship [28]—possibly due to an increase in active motor units [29]. PFM bioelectrical activity has been associated with hip muscle strength [16], but we did not find any studies that demonstrated such an association with HGS. In this study, we found that HGS was a moderately positive predictor of MVC and mean amplitudes of PFM contraction, suggesting that PFM function may be a good predictor of overall muscle strength [6,7].

The best indices of sexual function are found in women with better PFM functionality. The results of this study show that sexual function may affect the relationship between HGS and PFM functionality in the sample studied. The research conducted by De Luccas et al. [30] on PFM functionality suggests that women with weak muscles are more likely to have sexual dysfunction. Another study found that sexually active and orgasmic women had better PFM resistance than non-sexually active women [31]. Although the assessment of sexual function is complex due to its multifactorial nature (association with biological, sociocultural, and interpersonal factors), some studies have demonstrated the use of electromyography in this assessment. Omar et al. [32] investigated the possibility of using electromyographic measurement techniques to quantitatively assess female sexual function and objectively demonstrated that electromyography is a potential technique to quantify changes in female sexual function. In another study, Sartori [33] analyzed PFM contraction time (assessed with a perineometer) and electromyography; he found that PFM contraction time was significantly longer in women with orgasm, confirming the interaction reported in the study.

In addition, physical activity level affects the relationship between HGS and PFM function. A recent review [34] argued that there is more evidence to support the hypothesis that physical activity is a risk factor for PFM dysfunction, but no consensus was reached. It is also important to note that people with PFM dysfunction tend to be more sedentary (especially those with urinary incontinence) [35], as women with high levels of physical activity are more likely to have stress urinary incontinence. This could be related to the lack of pre-contraction of the PFMs or the delay of this mechanism during physical activity; this leads to a weakening of this muscle group and thus to PFM-related dysfunction [36]. Thus, regardless of the level of physical activity, women can be equally analyzed in terms of indirect assessment of PFMs by HGS.

In concordance with our findings, another study evaluating the impact of BMI on PFM function showed that this variable did not interfere with the pelvic floor [33]. However, another author argues that an increase in abdominal pressure may directly be associated with high BMI and central obesity [37], which causes continuous pressure on the PFMs and can therefore compromise the function of these muscles. In this aspect, the reason why our results differed may be the fact that our sample population had a mean BMI within the normal range.

This study presents innovative findings in the literature: it shows a great possibility of predicting the level of bioelectrical activity of PFMs using overall muscle strength; this may help in cases where vaginal palpation (an invasive, non-objective examination shown not to correlate with overall strength in this study) is not possible. These findings could serve as engines for directing the treatment and prevention of PFM dysfunction and improving the population’s quality of life. However, it is important to emphasize that PFMs have the potential to activate contraction of the rectus abdominis and adductor muscles [38] so that the muscles of the body act synergistically during dynamic activities.

## 5. Limitations of the Study

The use of electromyography needs to follow strict protocols during the detection, analysis, and interpretation of electromyographic signals. However, the recommendations of the International Society of Electrophysiology and Kinesiology concerning electromyography are not specific for PFM. Therefore, these recommendations should be included for evaluating the use of electromyography in publications to guarantee reproducibility and reliability for new studies. Another limiting factor was the use of state-of-the-art technology, which involved high research costs. However, we performed sample calculations to ensure a minimum and sufficient sample size.

## 6. Conclusions

We conclude that healthy young and middle-aged adult women show no differences in HGS and PFM functionality. Moreover, HGS is related to mean amplitudes, MVC, sexual function, and physical activity, suggesting that PFM function can be related to overall muscle strength. In addition, the study highlights the subjectivity of PFM assessment by vaginal palpation, which did not show important correlations in the sample studied.

## Figures and Tables

**Table 1:** Social and anthropometric factors in the different groups.

	Young Adulthood (*n* = 30)	Middle Age (*n* = 14)	t or Z or χ^2^	*p*-Value
**Age (years)**	24.3 ± 3.28	43.36 ± 7.28	−9.359	<0.0001 ^#^
**Race/color (%)**			2.598	0.458
Black	13.33	7.14		
Brown	60.00	64.29		
White	26.66	21.43		
Yellow	0.00	7.14		
**Scholarity (%)**			4.490	0.106
Elementary school	0.00	14.28		
Secondary school	50.00	42.86		
Higher education	50.00	42.86		
**Marital status (%)**			22.269	<0.0001 *
Single	86.66 ^▀^	14.28		
Stable union	0.00	14.28 ^▀^		
Divorced	13.33	71.43 ^▀^		
**Family income (%)**			0.496	0.920
<1 minimum wage	20.00	21.43		
>1 and ≤2 minimum wages	20.00	21.43		
>3 and ≤4 minimum wages	23.33	14.28		
>4 minimum wage	36.66	42.86		
**Height** (m)	1.60 (0.11)	1.56 (0.12)	−2.008	0.045 ^♰^
**Weight** (kg)	62.06 ± 10.27	63.92 ± 9.64	−0.554	0.583
**BMI** (kg/m^2^)	23.03 (4.31)	26.22 (4.06)	−1.933	0.053

Body mass index (BMI).^#^ *p* < 0.05 (2-sided) by unpaired t-test. ^♰^ *p* < 0.05 (2-sided) by Mann–Whitney U. * *p* < 0.05 (2-sided) by Pearson χ^2^. ^▀^ Significant values through the adjusted residual post-hoc tests with Z_crit_ = 1.96.

**Table 2:** Clinical variables in the groups: young adulthood (*n* = 30) and middle age (*n* = 14).

	Young Adulthood (%)	Middle Age (%)	χ^2^	*p*-Value
**Hormone replacement**	6.66	7.14	0.003	0.953
**Pregnancy**			25.611	<0.0001 *
0	83.33 ^▀^	14.28		
1	13.33	21.43		
2	3.33	42.86 ^▀^		
3	0.00	21.43		
**Abortion**				
0	0.00	7.14	6.267	0.044 *
1	3.33	21.43 ^▀^		
2	0.00	7.14		
**Active sexual life**	86.66	64.29	2.939	0.086
**Urinary disorders**				
Nocturia	33.33	35.71	0.024	0.877
Preventive urination	50.00	64.29	0.786	0.375
Urinary tenesmus	43.33	64.29	1.676	0.195
Urinary tract infection (last 3 months)	6.66	14.28	0.003	0.953
Enuresis	23.33	50.00	3.129	0.077
Urinary urgency	30.00	28.57	0.009	0.923
Urge urinary incontinence	6.66	14.28	0.670	0.413
Stress urinary incontinence	43.33	57.14	0.730	0.393
Dysuria	10.00	7.14	0.094	0.759
**Anal dysfunctions**				
Constipation (ROMA III)	40.00	42.86	0.032	0.858
Fecal urgency	10.00	28.57	2.461	0.117
Fecal tenesmus	36.66	21.43	1.022	0.312
Soiling	13.33	21.43	0.468	0.494
Fecal leakage	0.00	7.14	2.193	0.139
Hemorrhoids	13.33	7.14	0.363	0.547

* *p* < 0.05 (2-sided) by Pearson χ^2^. ^▀^ Significant values through the adjusted residual post-hoc tests with Z_crit_ = 1.96.

**Table 3:** Handgrip strength, level of strength and bioelectrical activity of the pelvic floor muscles, International Physical Activity Questionnaire (IPAQ) and Sexual Quotient—Female version (SQ-F) in the different groups.

	Young Adulthood (*n* = 30)	Middle Age (*n* = 14)	t or U or χ^2^	*p*-Value
**Handgrip strength**	53.27 ± 10.46	53.14 ± 17.01	0.025	0.980
**Power/Pressure**	3 (1)	3 (2)	−1.747	0.081
**sEMG**				
MVC (µv)	36.5 (18)	31 (25)	−1.152	0.250
Peak (0 to 10 s)	101.90 ± 14.56	101.38 ± 12.44	0.118	0.907
Peak (%MCV)	101 (16.75)	99 (20)	−0.053	0.958
Mean amplitudes (0 to 10 s)	69.77 ± 15.51	63.00 ± 17.34	1.213	0.239
Mean amplitudes (%MVC)	70.5 (15.75)	61 (26)	−1.350	0.177
**IPAQ (%)**			1.102	0.894
Active	40.00	35.71		
Irregularly active A	10.00	21.43		
Irregularly active B	16.66	14.29		
Very active	26.66	21.43		
Sedentary	6.66	7.14		
**SQ-F**	74 (22)	50 (43)	−1.640	0.101

Surface electromyography (sEMG); Maximum voluntary contraction (MVC); International Physical Activity Questionnaire (IPAQ); Sexual Quotient—Female version (SQ-F).

**Table 4:** Relationship of pelvic floor muscle functionality, Sexual Quotient—Female version (SQ-F), and self-declared physical activity with the handgrip strength in healthy women with interactions.

				Collinearity Statistics	
	Standardized Coefficients β	t	*p*-Value	Tolerance	VIF
**sEMG**					
Peak (%MCV)	0.338	1.582	0.123	0.399	2.503
Mean amplitudes (0 to 10 s)	−0.566	−2.220	0.033 *	0.281	3.555
Mean amplitudes (%MVC)	−0.068	−0.330	0.743	0.424	2.359
MVC (µv)	0.516	2.105	0.043 *	0.304	3.286
**Power/Pressure**	0.190	1.179	0.246	0.704	1.420
**SQ-F**	0.319	2.037	0.049 *	0.747	1.338
**Self-declared physical activity**	−0.442	−3.081	0.004 *	0.886	1.129

* *p*-Value < 0.05, multiple linear regression with backward elimination effects.

## Data Availability

Survey data is available at the link: https://docs.google.com/spreadsheets/d/197gFkrtn0DJsvW7cnLBN3mrUgJsE01b2dwkBHwThne0/edit?usp=sharing.
